# The Role of Ageing and Parenchymal Senescence on Macrophage Function and Fibrosis

**DOI:** 10.3389/fimmu.2021.700790

**Published:** 2021-06-17

**Authors:** Ross A. Campbell, Marie-Helena Docherty, David A. Ferenbach, Katie J. Mylonas

**Affiliations:** ^1^ Centre for Inflammation Research, Queen’s Medical Research Institute, University of Edinburgh, Edinburgh, United Kingdom; ^2^ Department of Renal Medicine, Royal Infirmary of Edinburgh, Edinburgh, United Kingdom

**Keywords:** macrophage, senescence, ageing, fibrosis, immunoageing, immunevasion, senolytic, senescence-associated secretory phenotype

## Abstract

In this review, we examine senescent cells and the overlap between the direct biological impact of senescence and the indirect impact senescence has *via* its effects on other cell types, particularly the macrophage. The canonical roles of macrophages in cell clearance and in other physiological functions are discussed with reference to their functions in diseases of the kidney and other organs. We also explore the translational potential of different approaches based around the macrophage in future interventions to target senescent cells, with the goal of preventing or reversing pathologies driven or contributed to in part by senescent cell load *in vivo.*

## Introduction

Ageing in humans is marked by a decrease in fitness over time with a simultaneous increase in mortality (1). With increasing age, the functions of key biological systems begin to decline, as shown by decreased nutrient sensing, stem cell exhaustion and cellular senescence (1). Due to advances in healthcare, human life expectancy has increased worldwide, with estimates of the average global population placing 1 in 9 people over 60, which is expected to rise to 1 in 5 by 2050 (2). As we age, the incidence of physiological dysfunction increases as well, and thus the risk of age-associated diseases such as chronic kidney disease (CKD), cardiovascular disease and type II diabetes (3) also increases. These diseases predispose individuals to developing additional pathologies and increase both morbidity and mortality. This calls for polypharmaceutical treatments and interventions to maintain quality of life (2), which come with the caveats of side-effects and cross-reactions and are therefore potentially detrimental to patient welfare. Because of these drawbacks, novel treatments need to be developed to target the causes of these co-morbidities to reduce the need for large amounts of medication.

This review aims to summarize the current understanding of the functions of senescent cells and macrophages, and their combined effect on fibrosis within tissues. Focus will be given to the kidney, supplemented by other relevant organ systems. Finally, we will summarize the challenges for future research, and potential avenues for translating research studies into therapeutic advances.

## Ageing and Disease

**Figure 1:**
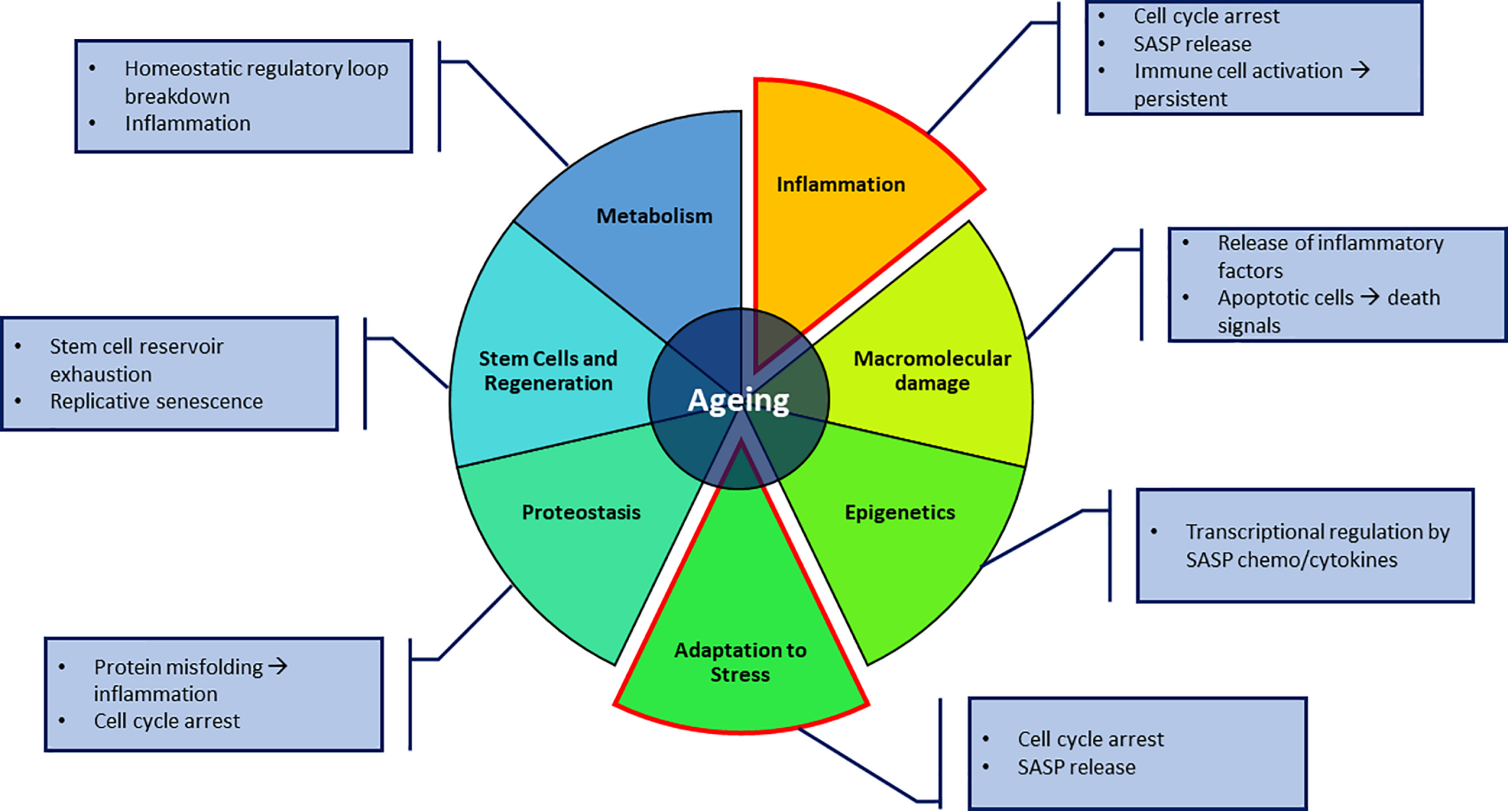
The seven pillars of ageing proposed by Kennedy et al. (4). Key aspects in this review focus on the adaptation to stressful environments in the form of senescence and macrophages, and the inflammatory effect it can have on its surroundings. Annotations to each pillar highlight possible contributions to direct senescence or producing a senescence-promoting environment.

The trans-NIH Geroscience Interest Group (GSIG) held a summit in 2016, focusing on the seven pillars of ageing, expanding on previously assessed hallmarks (1). These seven factors include metabolic changes, macromolecular damage, epigenetic changes, inflammation, adaptation to stress, stem cells and regeneration and changes to proteostasis (4), all of which share interconnected relationships (**Figure 1**).

Ageing is associated with progressive decline in function of multiple organ systems. Bone loss has long been associated with advancing age, with a reduced capacity to heal fractures (5), which is marked by a decrease in osteocytes with increasing age (6). The consequences of this are seen in the increase of non-traumatic bone fractures, with the frequency of these fractures increasing for persons aged over 60 (5).

Cardiovascular function is negatively impacted by age, as the arterial tree can thicken and stiffen (7). This trend, seen in both genders, and as measured by carotid-femoral pulse wave velocity (PWV), diverges at age 50 for men and women, with men having a steeper increase of PWV (7). This has key implications for certain diseases such as end-stage renal disease, as significantly higher ‘pulse-wave velocity’ (PWV) has been used to predict mortality in patients (8). This indicates certain aspects of ageing differ based on gender, suggesting a hormonal role contributes to the phenotype, however this is beyond the focus of this review and will not be discussed.

Chronic kidney disease (CKD) is relevant in ageing studies as it has been found that there are signs of premature ageing in CKD patients such as osteoporosis, poor wound healing and inflammation, leading to the proposal that CKD be included as a disease that displays traits usually associated with advanced ageing (9). The aged kidney is marked by up to 40% less renal blood flow in old *vs*. young male patients (10). Increasing donor age is associated with reduced transplant function after donation, even in the context of well-preserved pre-donation function (11). Overcoming these problems requires investigating the links between ageing and physiological dysfunction, which is the greatest risk factor for the diseases listed previously.

Ageing is associated with decline in cellular functions, including the phagocytic clearance of cells. This has been shown using *in vitro* mouse models in which serum from aged mice (24 months old) was added to cultures of macrophages, which resulted in decreased levels of phagocytosis (12). In addition to this, direct studies of clearance of apoptotic skin cells (induced by UV B irradiation) showed a higher level of apoptotic keratinocytes compared to younger mice indicating reduced phagocytic activity. Of note, in aged mice with reduced phagocytosis of apoptotic cells, renal autoantibodies developed along with complement deposition within the kidney, findings consistent with the development of autoimmunity (12). Macrophage numbers were not significantly reduced or increased in older mice compared to younger mice in these studies and therefore, a decrease of macrophage phagocytic activity is implicated. This shows that the persistence of apoptotic cells has a detrimental effect on normal tissue function, particularly the kidney (13). However, when macrophage phagocytosis was assessed *in vitro*, there was no difference between young and old derived, indicating systemic factors may modulate the potency of macrophage phagocytosis (13).

‘Gerontology’ focuses on ageing and older adults, whereas ‘geroscience’ emphasizes the overlap of normal ageing and chronic disease. The geroscience hypothesis predicts that the targeting of the suspected drivers of ageing will also mitigate the main risk factors of multiple chronic diseases (4). This would have the potential to increase the health span of individuals. An example of this is caloric restriction (CR), and has long been known to have pro-longevity properties as first seen in *C.elegans* (14). This has since been investigated in mouse models, which showed an increase in longevity, decreased cancer incidence and a rejuvenated immune system through intermittent fasting (15). Data for controlled calorie consumption in humans showed a limited impact from calorie restriction (16). However, a meta-analysis of clinical studies indicated increases of fibrinolytic activity, to degrade fibrin deposits, improving prognosis for cardiovascular disease patients (17). This indicates at least partial benefits when applied to humans. However, this may come at a cost, as recent studies in grey mouse lemurs (*Microcebus murinus*) indicated that moderate caloric restriction (30%), accelerated grey matter atrophy (18). Despite this, lifespan was increased (50% median increase), with a decrease in nephritis as cause of death and cognitive function comparable to controls. Further studies are warranted to make comparisons to humans who have higher-level reasoning capacities that may not be properly assessed with animal models.

## Senescence

Senescence involves irreversible arrest of the cell cycle (19) and was first observed by Hayflick and Moorhead (20). Multiple factors can stimulate senescence in a cell, including DNA damage, chronological ageing in the form of telomere shortening and cell stress from chronic conditions (19, 21, 22). This is marked by increased expression of cyclin dependent kinase inhibitors p21^CIP1^ and p16^INK4a^ (23, 24) that drive cell-cycle arrest. Despite the impact of external stress, senescent cells remain metabolically active with an altered secretome of cytokines, chemokines and proteases that have the capacity to modulate the activity and functionality of surrounding cells. This is called the senescence-associated secretory phenotype (SASP) (25). These include, but are not limited to, interleukin-8 (IL-8) (26), TNF-α (27), IL-6 (28) and IL-1α (29). Alone, none of these markers are definitive for senescence. Combinations of other markers such as senescence-associated β galactosidase (SA-β-Gal), a marker of increased lysosomal activity, γH2AX which is a marker of double DNA strand breaks and the DNA damage response, along with markers for cell-cycle checkpoints (p21^CIP1^, p16^INK4a^) (30) are used to infer senescence of cells.

### Acute *vs* Chronic Senescence

Acute senescence is a tightly regulated process seen during wound healing (31, 32), in embryogenesis (33, 34), and in protection from cancer (35). Wound healing is a tightly regulated process of inflammation, infiltration, and proliferation, with specific proteins driving senescence of particular cells. The CCN1/CYR61 matricellular protein, is involved in this process and can induce senescence in the fibroblasts at site of wound repair (32). Mouse studies in which the CCN1 locus was replaced a mutated sequence showed that mice lacking the active form of this gene had faster wound healing but greater levels of fibrosis (scarring) and lower levels of senescence markers including SA-β-Gal and p16^INK4a^ (32) compared to WT. As the exogenous addition of CCN1 to cutaneous wounds of mutated mice resulted in the increase of *Mmp2*, *Mmp3*, and *Mmp9*, and reduced expression of *Col1a1* and *Tgfb1* (32), this indicates a role of senescence in limiting excessive fibrosis in wound healing, where fibrosis could become detrimental. The beneficial role of senescence in acute wound healing is supported by studies using genetically-mediated depletion of senescent cells - where senescent cell removal resulted in a delay in skin wound closure (31).

Chronic senescence is marked by senescent cells that accumulate with age and chronic diseases, such as pre-cirrhotic fibrosis of livers (36, 37) and chronic kidney disease in which senescent cell accumulation correlates to increased disease severity (38) Accumulation of senescent cells also contributes to radiation toxicity from radiotherapy (39, 40) and chemotherapy (39). It is believed that chronically senescent cells become problematic when they are not destroyed and cleared by immune cells, such natural killer (NK) cells and macrophages (41), and continue to generate signaling molecules that systemically and locally affect the normal function of cell (19).

Senescent cells that accumulate with age, and their associated SASP, can alter the functions of other cells of an organism (42–44). This ties in closely with the geroscience hypothesis (4). If ageing is viewed as a disease, certain drivers have been identified. For example, the expression of p16^INK4a^ has both short and long term impacts, as it prevents cancer by triggering senescence, the cost of which is that ageing is promoted (45). Selective p16^INK4a^ ablation ameliorates some ageing phenotypes, increasing production of T-cells and increases antigen-specific immune responses, but causes an increased risk of cancers such as high-grade B-cell neoplasms (45). Defense against cancer initiation is one of the key roles of senescence, with oncogenes such as RAF or BRAF causing oncogene-induced senescence (35). The advantage of senescence in this case would be the continued survival of an organism with gradual decline from ageing versus the more imminent death of the organism due to increase in cancer incidence, an example of antagonistic pleiotropy. This highlights the need for senescence as a protection mechanism, but a need to remove senescent cells after they have fulfilled their protective role, to prevent the detrimental impact of exposure of the SASP to healthy tissues and organs.

## Senescence in the Kidney

### Senescence With Age/Injury

When the kidney undergoes damage, the resident cells including renal tubular epithelial cells, endothelial cells, podocytes, T-cells (46) and macrophages (47) produce paracrine signaling factors that affect resident tissues, termed the chronic kidney disease (CKD)-associated secretory phenotype (CASP) (48). The parallels between the CASP and the commonly accepted SASP may be due to the presence of senescent cells within the dysfunctional kidney, as many cytokines of the SASP are also found in the CASP such as IL-8, TNF-α and IL-6 (48). This would suggest that any comparisons of the secretory phenotypes should be separated between senescent cells and the other cell types of the renal system as this may reveal other cell targets for therapeutics. This would help determine if SASP is driving the cell behaviors in the tissue or if they are independent.

Age has been shown to be a key contributor to progression of acute kidney injury (AKI) into chronic kidney disease (CKD) due to an inability to fully recover from an acute insult (49–51). Age can also be detrimental to transplantations, including kidneys, in which mice deficient in p16^INK4a^ displayed fewer senescent cells, with an increase in proliferative rates of tubular cells, all leading to significantly better survival of donor mice (52). Studies using bilateral renal ischemia-reperfusion to induce AKI in young (8-10 weeks) and ageing (46-49 weeks) mice showed an increase in fibrosis by picrosirius red staining and immunolocalization of cellular fibronectin, collagen III and collagen IV in the older mice (49). This correlates to higher levels of p53 and p21^CIP1^ expression as well as SA-β-Gal staining in the older mice. Less fibrosis and tubular atrophy was seen in p16^INK4a^ knock-out mice after ischemia-reperfusion injury (52) and in addition to this, the deletion of senescent cells in old (18 – 24 months) and prematurely aged mice, resulted in a better prognosis (53). Whilst activation and proliferation of fibroblasts can be a key wound-healing response to injury, excessive fibrosis can also be detrimental to physiological functions across multiple organs including the heart, kidney, and liver.

### Senescence in Other Organs

Liver pathologies can also be made worse by the presence of senescent cells as has been reviewed (54). Chronic injuries in the liver can lead to fibrosis, and may develop into cirrhosis, but may be resolved by several endogenous mechanisms, one of which is the CCN1 protein which induces senescence of hepatic myofibroblasts (55). However, this has also been shown to activate DNA damage response (DDR) pathways and p53 by engaging integrin α_6_β_1_ which generates the production of reactive oxygen species (ROS) which stimulates the DDR, activating the p53-p21^CIP1^ dependent pathway of cellular senescence (32, 55, 56). These acutely senescent cells display anti-fibrotic transcriptional programs, resolving potentially dangerous fibrosis of the liver (55). This also demonstrates the complex and contextually significant role of senescent cells in the body, which can be beneficial in injury resolution and wound healing, and must be taken into consideration with any systemic therapies that target senescent cells.

Senescent cells are found in multiple tissues of the body, including the bones and brain. For example, an increase in the number of senescent astrocytes are found in cadaveric Parkinson’s disease patients compared to normal control tissues, along with an increase in SASP markers IL-6, IL-1α and IL-8 (29). In mice, clearance of senescent [glial] cells of the brain has been shown to reduce the accumulation of hyperphosphorylated tau aggregates in the dentate gyrus (responsible for memory formation and cognition), that has been linked to cognitive decline (57) and Alzheimer’s (58). Similarly, clearance of senescent cells that accumulate in osteoarthritis produced a pro-regenerative environment as marked by subchondral sclerosis and osteophyte formation similar to controls (59). Inhibition of SASP factors generated by senescent cells, such as the profibrotic TGF-β, has also been shown to improve regeneration in the liver (60).

## Clearance of Senescent Cells by the Immune System

When senescence is induced, these cells need to be cleared from tissues to prevent damage to the surrounding cells. The SASP generated by senescent cells is able to promote immune clearance of the senescent cells (61). The SASP potency can be modulated by the epigenetic regulator BRD4 (Bromodomain-containing protein 4), as when this was knocked down, secreted SASP factors in *in vitro* cultures were reduced (62). *In vitro* experiments also showed that conditioned medium from senescent cell cultures induced senescence in naïve cells, halting proliferation and upregulating SA-β-Gal activity, whereas conditioned medium from BRD4-inhibited cultures had significantly reduced capacity to transmit senescence to naïve cells (62).

Senescent cell destruction within tissues can be carried out by natural killer (NK cells). One of the first instances in which this was reported was in a study in which p53 was activated in liver carcinomas using RNAi, resulting in rapid tumor regression, and inhibition of NK cells showing significantly delayed tumor regression compared to controls (63, 64). By Day 8 of p53 senescence induction, infiltration of leukocytes including NK cells was observed in mouse kidneys by immunohistochemistry, with detection of NK-specific transcripts (*Klrb1* and *Klrd1*) upregulated in tumors suggesting NK infiltration because of the presence of senescent tumor cells (63).

NK cells are not the only immune cell involved in the clearance of senescent cells. Macrophages can be recruited by numerous factors, including SASP factors and the secretome of NK cells. NK cells can produce interferon gamma (IFN-γ) upon interaction with senescent cells (65) which acts to recruit macrophages (66). In addition to this the induction of tumor senescence can lead to an increase in oxidative stress, as shown in breast cancer due to protein acetyltransferase dysregulation (67). This led to an increase in chemoattractants CCL2, CXCL1, CXCL16 and IL-8, which recruit NK cells and macrophages, causing clearance of senescent tumors (67). This is matched in other organs; for example the liver where senescent hepatic myofibroblasts, produce CCL2, attracting CCR2^+^ macrophages to the liver to clear pre-cancerous cells, demonstrating a protective role for senescence in the liver (68). Together this shows the orchestration of immune cells by signaling of senescent cells to exert a protective anti-cancerous role, with the roles of macrophages in senescence being explored later.

## Macrophages

### Functions

Macrophages have a diverse range of functions, including tissue homeostasis, immune surveillance, resolving cutaneous wound healing (69), clearance of red blood cells (70, 71) and even supporting embryogenesis (72). One of their primary functions as ‘big eaters’ is to phagocytose cells, such as apoptotic cells [efferocytosis (73)] and senescent cells (33). One component of the macrophage lysosome is DNase II which degrades the DNA of engulfed cells (74). Mouse experiments in which DNase II was knocked out, showed undigested DNA within the lysosomal compartment of macrophages, leading to activation of the immune system as marked by increases in IFN-γ and more moderately, TNF-α (74). This indicates that incomplete phagocytosis can be pro-inflammatory. During inflammation of the body, macrophages can be recruited by chemotactic factors secreted by neutrophils to aid in inflammation resolution. This is useful in host defense against pathogens in which macrophages phagocytose apoptotic neutrophils (75) which promotes inflammation resolution (76).

The ability of macrophages to robustly clear senescent cells from the tissues of the body diminishes with age (77). One proposed explanation for this is for macrophages to be able to acquire senescent-like properties, termed “senescent associated macrophages (SAMs)”. A possible cause of this may be due to the ability of the SASP of senescent cells to induce senescent features in macrophages such as SA-β-Gal positivity (28, 78). However, as there is inherent β-galactosidase activity in macrophages due to their lysosomes (79), this cannot be an accurate marker to definitively class macrophages as senescent.

### Macrophages in the Kidney and Models to Dissect Senescent Cell–Macrophage Interactions

Resident macrophages of the kidney arise from three separate sources, the C-Myb independent yolk-sac EMP-derived, fetal liver C-Myb dependent EMP-derived and finally, hematopoietic stem cells (HSC) as comprehensively summarized (80). Macrophages in the adult kidney can be separated by markers into distinct populations, which may result in them displaying differing responses to stimuli.

Circulating monocytes can populate the kidney and mature to macrophages to continue to provide immune and injury response, but these are not precise analogues to the long-lived kidney resident macrophages (KRM) (81). Models have been developed to study the effect of senescent cells in the kidneys of young mice (82). These models utilize *Pax8* which is a transcription factor involved in the formation of the kidney tissues in mice, with human homologues (83). Conditional excision of *mdm2* is driven by *Pax8*; *mdm2* codes for MDM2 which negatively regulates p53, promoting stabilization and degradation by ubiquitination, promoting senescence induction (84). The *Csf1r^ΔFIRE/ΔFIRE^* (FIRE) mouse has recently been developed, and shows robust elimination of resident F4/80^high^ macrophages in multiple tissues of interest including the kidney (85). This was achieved by the removing the super enhancer (*fms*-intronic regulatory element) located in the second intron of the *Csf1r* gene (85). This improves on the limitations of the *Csfr1^-/-^* macrophage deficient mouse model which showed lack of bone remodeling (osteopetrosis), resulting in deformities during development, and reproductive defects, resulting in lower pregnancy rates (86). In addition to this, the niche population is not repopulated by circulating monocytes (85), which suggest the alteration of the niche-cell interaction by the removal of the CSF-1 receptor from F4/80^+^ cells (85).

These models will provide information of the effects of senescent cells in young animals and their effects on macrophages in an *in vivo* setting. These two models may provide insight into how different macrophage populations interact with senescent cells of the kidney, for instance in the absence of resident F4/80^high^ renal macrophages, and in mice exposed to chronically senescent cells from an early age.

## Macrophage Plasticity

**Figure 2:**
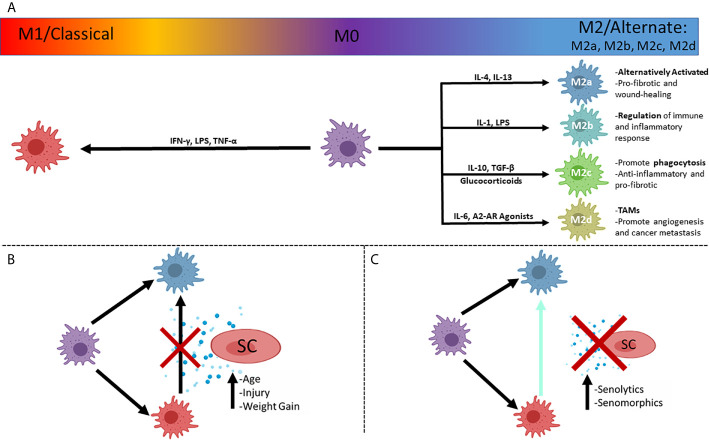
The simplified polarizations of macrophages and potential interactions with senescent cells. **(A)** Macrophages can be in an unpolarized M0 state, and polarize to the classical inflammatory M1 state, or the alternately activated, pro-reparative M2 state, with subcategories of M2 activation that dictates function, with prominent drivers listed. **(B)** Plasticity of macrophages allows a shift in polarized states from M1 to M2 that can be inhibited by the secretosome of senescent cells (SASP), associated with injuries to organs and increasing age. **(C)** Inhibition of polarization shift can be ameliorated with pharmaceutical compounds ultimately minimizing the effect of the SASP. SC, Senescent cell; TAMs, Tumor Associated Macrophages.

Macrophages display phenotypes adapted to their various roles and can be classified very simply as M1 (pro-inflammatory) or M2 (pro-regenerative) states based upon cell surface markers, synthesis of specific factors and biological activities. This was first suggested by Mills and colleagues, based upon the Th1/Th2 paradigm (87) and represents extremes of a spectrum of polarization states (88) (**Figure 2**). M1 macrophages generate inflammatory cytokines (89) in response to microbial infection or inflammation, such as IL-6 (90), nitric oxide (NO) (91, 92), TNF-α, IL-1β, IL-12 and IL-23 (93, 94). Inflammatory monocytes are recruited to sites of acute and chronic kidney injury, with the SASP-associated CCL2 promoting the accumulation of cytotoxic and pro-inflammatory M1 (classically activated) macrophages (95, 96).

After injury, pro-reparative or ‘alternatively activated’ M2 macrophages can be induced by exposure to Th2 type cytokines, such as IL-4 and IL-13 (97, 98) or phagocytosis of apoptotic cells, with transition from M1 to M2 polarization seen in successful resolution of inflammation by the production of mitogenic and pro-survival signals that promote renal repair (88, 99, 100). Failure of macrophages to switch from a pro-inflammatory state to the pro-reparative state has been implicated in chronic diseases, including chronic kidney disease due to the excessive fibrosis caused by the presence of a proinflammatory environment (49). M2 macrophages have anti-inflammatory roles, secreting cytokines such as IL-10, and express markers such as CD206, Ym1, CD163, CCL1, CCL18, FIZZ1, arginase1 (Arg1) and chitotriosidase genes (101–103). In the lungs it has been shown that M2 macrophages are able to suppress inflammation secretion of factors that affect the behavior of surrounding cells (103).

M2 macrophages are crucial to wound healing, with their functions including clearance of debris, activation of regulatory T-Cells, suppression of inflammation, reduction of neutrophil infiltration and antagonism of M1 macrophage functions (104). M2 macrophages can be further subdivided into M2a,b,c and d types (**Figure 2**), with M2a being the most commonly described and referred to as alternatively activated (IL-4 induced) (100). M2a are activated by IL-4 and IL-13 while M2c are activated by IL-10 and TGF-β, and glucocorticoids (105). M2a (wound-healing macrophages) are profibrotic, secreting TGF-β, insulin-like growth factor (IGF) and fibronectin (106). M2c potently induce regulatory T-cells, which aid in protection from renal injury (104, 107). It has been shown, using mouse renal models, that both M2a and M2c polarizations can be robustly induced in aged mononuclear cells *in vitro*, whereas polarization was limited *in vivo* in IRI models when compared to young mice (108). This suggests that the intrarenal microenvironment of aged mice after IRI has a greater negative impact on macrophage polarization than the ageing of the bone-marrow derived monocytes (108). M2c subtypes also have strong anti-inflammatory and pro-fibrotic functions due to the production of IL-10 and TGF-β, respectively, with high expression of Mer receptor tyrosine kinase (MerTK) for efficient phagocytosis of apoptotic cells (104, 106). M2b (regulatory macrophages) are activated by IL-1, LPS (105) and secrete both pro-inflammatory factors (IL-1β, IL-6, and TNF-α) as well as the potent anti-inflammatory IL-10, due to their roles in the regulation of inflammation and immune response (88, 106). M2d (tumor-associated macrophages – TAMs) are activated by IL-6 and A2 adenosine receptor (AR) agonists (105, 106), and represent a more detrimental class of M2 macrophage as they contribute to angiogenesis (by release of vascular endothelial growth factor – VEGF) and cancer metastasis (109, 110).

As with M1 macrophages, niche context can also influence M2 functions, as increased fibrosis (collagen I, II and III deposition) is seen in injured kidneys due to the signaling effects of macrophages on resident kidney fibroblasts (111). To some extent, macrophage origin can also affect polarization capacity, as the M2a subtype derived from bone marrow was more likely to switch to an inflammatory phenotype, than M2a cells derived from the spleen (104). Macrophages are not terminally differentiated and retain the ability to switch to other phenotypes. As well as M1 macrophages having the ability to switch to a more pro-repair phenotype (99), M2 macrophages can be induced to adopt proinflammatory features to enable microbial killing (112).

### Macrophages and Fibrosis

In the kidney, with increased ageing, fibrosis can be driven by the upregulation of the Wnt/β-catenin signaling and renin-angiotensin system (RAS) pathways, shown by an increase of fibronectin and picrosirius red staining (113). This was validated by the experimental overexpression of Klotho which acts as an antagonist of Wnt/β-catenin, resulting in diminished renal fibrosis, preservation of mitochondrial mass and reduced production of reactive oxygen species (ROS) (113). Levels of Klotho decrease with age in mice (over 12 months), correlating to increase in fibrosis and aging markers. This indicates that potent and selective inhibitors of the Wnt/β-catenin pathway could provide a useful therapeutic for age-related fibrosis as well as fibrosis caused by the presence of senescent cells due to injury in the kidney.

However, fibrosis may be caused by other mechanisms, such as macrophages. Pro-reparative (“M2”) macrophages have the potential to accelerate tissue repair, but if they remain persistently activated, or are continually recruited, they may contribute to chronic fibrosis (114). This is due to secreted cytokines from macrophages, such as TGF-β which simultaneously has anti-inflammatory and profibrotic activity (114).

Tissue fibrosis may be impacted by the effect of ageing on macrophages, which undergo changes in secretory production. Importantly the anti-inflammatory IL-10 cytokine is decreased (115). This has important implications for tissue function as IL-10 is also anti-fibrotic by inhibition of pro-fibrotic molecules such as TGF-β (116). Recent therapeutic research has focused on the potential of utilizing IL-10 for its potent antifibrotic properties (117). This highlights an important decline in a key M2 macrophage signaling molecule, demonstrating a decrease in potency with host age and leading to tissue environments more permissive to fibrosis.

### Macrophages in Kidney Injury

When the kidney is injured, proliferative monocytes are recruited and infiltrate the kidney to the site of tissue damage (118). This occurs after IRI (119) along an IL-6 chemotaxis (120), among other signaling factors. Monocyte infiltration begins early after injury, as shown by increased F4/80 staining at day 1. These monocytes migrate towards injured tubules of the outer medulla and mature to macrophages (121). Macrophages enhance pro-inflammatory damage caused by injury, as depletion of macrophages can be beneficial at early timepoints, as marked by a decrease in in blood urea nitrogen (BUN). However, they are also needed for resolution of inflammation and tissue repair, as their depletion (by liposomal clodronate) is detrimental at later (72 hour) timepoints at which recovery begins (13). This affect appeared to be due to the inflammatory environment of the kidney and how it changes over the first 72 hours of injury, in which pro-inflammatory cytokines such as CCL2 were upregulated in the first 24 hours, and anti-inflammatory IL-10 rose in later stages (13). This indicates that macrophages involved in kidney injury have functions that are dependent on the local cytokine signals, which affect their polarization, and show a beneficial effect when depleted at early inflammatory timepoints and a beneficial effect when re-administered at later timepoints. Depletion of macrophages by clodronate liposomes has been shown in other studies, delivering functional protection and reduced acute tubular necrosis (122). Genetic diphtheria toxin (DT)-mediated depletion of CD11b-DTR mouse macrophages did not give a protective effect compared to controls, possibly due to clodronate having a minimal effect on resident CD11b^+^ populations (122). This suggests that partial depletion of mononuclear phagocytes at earlier timepoints has a cytoprotective effect.

After renal IRI, M2 macrophages are present at later timepoints of injury repair (~day 3), coinciding with peak cell division of tubular cells (121). Gene expression assays by qPCR showed that macrophages (fluorescence associated cell sorted for F4/80^+^) in the injured kidneys displayed a concurrent decrease of iNOS and increase of Arg1, markers of pro-inflammatory/classical and alternatively activated macrophages respectively (121). To verify a transition of macrophage states from M1 to M2, and not polarization of infiltrating monocytes, bone marrow monocytes were labelled with PKH26 and exposed to interferon gamma (Ifn-γ) to stimulate iNOS-positive, pro-inflammatory M1 polarization, and injected 3 days post-IRI. PKH26 labelled cells collected at day 5 displayed downregulated iNOS expression and increased CD206 (mannose receptor), a marker for M2 polarization, suggesting their polarization altered in response to their environment (121).

Most of these studies have been carried out on young mice. However, the presence of senescent cells can impact macrophage phenotype. For instance, inhibition of the SASP cytokine TNF-α in human macrophages derived from peripheral blood mononuclear cells (PBMCs) induced a shift in polarization of macrophages to an M2 phenotype from an M1 phenotype and decreases the secretion of pro-inflammatory cytokines (TNF-α, IL-6 and IL-12) (123). Further experiments showed an increase in phagocytosis of M1 macrophages. Taken together, this indicates that through SASP signaling, senescent cells can reduce phagocytic activity of macrophages and inhibit the transition to pro-reparative M2 macrophages, leading to a persistence of pro-inflammatory M1 macrophages. How the presence of senescent cells with ageing might affect the roles of macrophages in inflammation and tissue repair after injury of the kidney and other organs warrants further experimental investigation.

## Senescence Induction in Macrophages

The concept of “macrophageing” was first introduced in 2000 (78, 124, 125) and suggests that macrophages are induced into becoming senescent in response to signaling from surrounding cells. This has been a controversial subject in previous years, as although macrophages may display upregulation of senescence associated markers such as p16^INK4a^ and SA-β-Gal (126) they remain proliferative indicating that they have not acquired “true” senescence (127). Evidence indicates that these markers that emerge are contextual in macrophages and may in fact be indicative of their polarization (128). Recent research has provided more evidence for senescent macrophages, as shown by significant upregulation of both p16^INK4a^ and p21^CIP1^ cell-cycle checkpoints in a mouse model of DNA damage repair deficiency (129). In addition to this, macrophages as sorted by F4/80^+^ and CD11b^+^ markers showed significant upregulation of SASP components, including TNF-α, IL-6 and IL-1β as compared to littermates, in which the coding sequence for DNA repair protein ERCC1 is not excised (129). This is matched by significant increases in p16^INK4a^ and p21^CIP1^ in the bone marrow compartment of the transgenic mice, compared to 2 year old wild-type control mice and un-induced controls, with an increase in the pro-inflammatory IL-6 accompanying this. This field is constantly being re-interpreted as new evidence is produced, and it is beyond the scope of this review to advocate for or against senescent macrophages, and will focus on the role(s) of senescent cells upon macrophages and their ability to function.

## Immunoageing and Immunevasion

Ageing of the immune system is marked by a chronic level of systemic inflammation, which contributes to the pathogenesis of age-related diseases called ‘inflammaging’ (124, 125). This can be seen in monocyte/macrophage populations of the body. The peripheral blood monocytes that in some cases invade tissues and supplement resident macrophages are reduced with age (130). The macrophages of the lungs (alveolar macrophages) have shown lower levels of phagocytosis of apoptotic neutrophils (75). This can lead to increased susceptibility to infections such as influenza, resulting in a higher mortality in mouse studies (75). In the brain, phagocytosis of amyloid-beta is reduced in multiple populations of blood monocytes (131).

The dysregulation of macrophage functions with age may be linked to low levels of innate immune activation as marked by sCD163 and CXCL10 (132). It was also found that age resulted in higher levels of inflammation as shown by an increase in TNF-α of aged monocytes compared to young human peripheral blood monocytes (132). Higher levels of inflammation are also seen in kidney macrophages of aged mice, with aged CD73^+^ kidney mesenchymal stromal cells upregulating *Ccl2* with a higher proportion of CCR2^+^ macrophages detected (95). This indicates an increase in pro-inflammatory macrophages with age. This is made more detrimental by the presence of senescent cells (**Figure 2**) that can cause macrophages to show ‘senescent traits’ (78), losing potency as shown by the decrease of IL-10 synthesis by “M2” macrophages (115) and a significant reduction in their capacity to phagocytose (133).

Macrophages of the spleen upregulate p16^INK4a^ expression up to 20-fold in response to ionizing radiation, which is in stark contrast to the 4-fold upregulation seen in T-cells (134). This indicates a higher sensitivity of macrophages to ionizing radiation, as shown by a sharp decrease in absolute cell number, which is rescued by targeted depletion of p16^INK4a^ cells. This would also indicate that immunosenescence induced by the SASP of splenic cells partially models the decline of immune system potency with age explained by inflammaging (78) and demonstrated by a decrease in phagocytosis with age (133). Given the key roles of macrophages in tissue repair, this higher sensitivity to the SASP components could be an explanation as to why senescent cell burden is such a detrimental component of the ageing soma and the role of decreased immune function has been shown to accelerate senescent burden (135). Despite rescue of cell number by p16^INK4a^ cell depletion, phagocytic activity measured by uptake of a fluorescent substrate, was diminished. It remains possible that there exists a spectrum of senescence due to the multiple components that can contribute to the phenotype, as senescence markers such as p16^INK4a^, p21^CIP1^, and SASP components (IL-6, IL-1α) can be present but well-established markers such as SA-β-Gal can be absent (134).

CD47 is a membrane protein recognized as an inhibitory signal of phagocytosis or a “don’t eat me” signal, first discovered in mice injected with red blood cells from CD47-null mice, in which the CD47 deficient red blood cells were rapidly cleared by splenic macrophages (136). As macrophages abundantly express the SIRP1α receptor, which binds to CD47, and depletion of splenic macrophages protected CD47^-/-^ red blood cells when injected into wild-type mice, this suggests the CD47-SIRP1α axis represents a method of evasion of immune clearance and CD47 as a “marker of self” (136). CD47 is overexpressed in cancers (137), and senescent cells upregulate expression of CD47 (138), including in human renal proximal tubular epithelial cells. This may allow cancers and senescent cells to appear as “self” to the immune system and escape clearance. As previously mentioned, senescent cells have roles in development (72), and it is possible that their ability to upregulate CD47 is an example of antagonistic pleiotropy (139), in which senescent cells need to avoid clearance during development and therefore upregulate CD47, an ability that becomes detrimental in later life as senescent cells persist and generate SASP.

Other immune cells are affected by age. The number of activated, CD56^bright^ NK cells that produce cytokines decreases with age in humans (>60 years) (140). *In vitro* studies have shown that senescent cells are able to escape by NK cell cytotoxicity by upregulation of HLA-E (MHC I) which can bind inhibitory receptors on NK cells (41). In addition, activating ligands for the NKG2D receptor such as MICA can be cleaved from the surfaces of senescent cells by matrix metalloproteases (MMPs) (141). These free MICA ligands are able to bind to the NKG2A of NK cells and inhibit binding to target cells, with a reversal of this evasion shown by a broad-spectrum MMP inhibitor (141).

## Therapeutic Interventions to Tackle Detrimental Effects of Chronic Senescent Cells

### Macrophage Therapeutics

As macrophages have been shown to be involved in kidney injury, with their removal at early timepoints appearing beneficial (13, 122), controlled depletion of macrophages may be of therapeutic interest. However, the methods used in these mice studies may not effectively translate to humans, and there may be off-target effects, depleting other macrophage populations. This could be deleterious in some settings, for instance if a patient was to suffer a myocardial event (142), and could disrupt normal homeostasis of red blood cell turnover by depletion of splenic macrophages (136).

Tailored genetic therapies are another possible option. Macrophages that have had their SIRPα receptor knocked out by CRISPr-Cas9, caused an increase of phagocytosis of human osteosarcoma cells by a factor of 4 (143). This study did not investigate phagocytic capacity of senescent cells, but logically as the macrophages are the cells being edited, senescent cells would also be more likely to be phagocytosed due to the lack of the SIRPα on the macrophages. Again, this is limited by its proof-of-principle in experimental settings, using human cell lines.

An alternative to time-consuming tailored healthcare, is the administration of humanized anti-CD47 antibodies (144). This study showed that the AO-176 antibody bound to human CD47, resulting in increased tumor cell death. An advantage of this is that the AO-176 antibody had minimal affinity for red blood cells, a common drawback of previous CD47 antibodies (144). Further investigation of whether this anti-CD47 antibody has any anti-senescent properties is warranted.

### Interventions to Improve Macrophage/Senescent Cell Clearance

#### Klotho

The kidney accumulates senescent cells with age, with mouse studies in which targeted depletion of senescent cells led to a decrease in age-associated pathologies in the kidney (43). Senescence is regulated internally by different mechanisms, one of which is the *Klotho* gene. ICGN (‘Institute of Cancer Research’- derived glomerulonephritis) mice transgenic for the *Klotho* gene had an average survival of 70% compared to ICGN controls (30%) (145). This overexpression of *Klotho* also led to a decrease in senescence associated SA-β-Gal, an established marker of senescence. Together, these indicate that *Klotho* positively affects survival, possibly by reducing senescent burden. *Klotho* knock-out mice have an increased number senescent cells (146), and as human levels of *Klotho* decline with CKD (147) it may be beneficial to increase soluble KLOTHO levels in patients. This repression of accelerated senescence, which is found with glomerulonephritis, preserves renal function and improves survival (145).

#### Senolytics and Senomorphics

**Figure 3:**
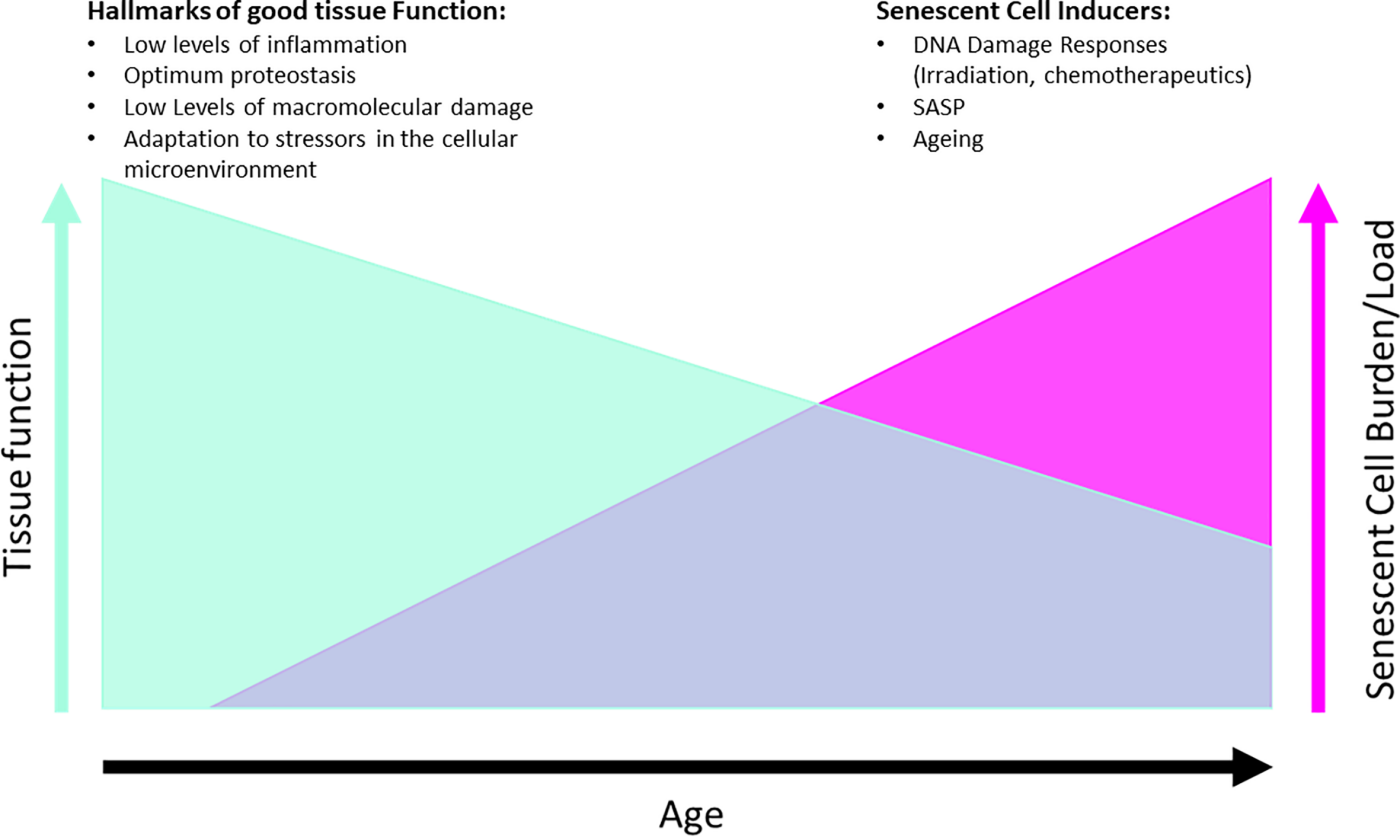
Hypothesis of effects of senescent cell removal therapies on tissue function, with examples of each of different hallmarks and drivers listed for both conditions. Graphs are not directly proportional as mitigating factors can alter the tissue functionality such as diet, exercise, disease.

Senescent cells have been shown to accumulate in age and in disease in both human and animal tissue and crucially, their clearance in animal models is safe and has been shown to improve health span (43) and organ function (39, 53). It has become of therapeutic interest to remove senescent cells, operating under the hypothesis that removal of chronically senescent cells will have a positive impact on local and systemic tissues (**Figure 3**). Drugs that accomplish this are called senolytics and treatments that target senescent cells are called senotherapies.

Recent studies have focused on small molecular compounds to inhibit the pro-survival mechanisms of senescent cells, such as ABT-263 (Navitoclax) and ABT-737 (148) which inhibit Bcl2/w/xL (39). ATB-263-induced elimination of senescent cells improves prognosis after IRI injury in mice as seen by decrease in fibrosis and ongoing injury, with increased levels of regeneration and better kidney function (53). Human trials of ABT-263 have revealed that the onset of thrombocytopenia (abnormally low platelet counts) may limit the dose/timing of ABT-263 administration to patients (149). Further research is required to determine if ABT-263 can be used as a senolytic in different settings, such as organ rejuvenation in transplants.

It has been shown that the senolytics, dasatinib and quercetin (D+Q) can be safely used in humans (150, 151). D+Q have a different target to ABT-263, and cause depletion of macrophages, with early studies showing a decrease of macrophage number per adipocyte by 28% (150). The control of senescent cell clearance by pharmaceuticals proves crucial, as inhibition of acute senescence present in wound healing (31, 32), could be disruptive to natural wound-healing processes that animal models/patients may be undergoing. This same study demonstrated that common markers for senescent cells, namely p16^INK4a^/p21^CIP1^ expression and β-galactosidase activity at pH 6.0, were decreased post senolytic treatment, but this can’t be attributed to Langerhans cell clearance or macrophage recruitment (150).

It has been proposed that noncoding RNAs (ncRNAs) may have a role in the regulation of SASP factors generated by senescent cells (152). Exogenous administration of ncRNAs capable of downregulating SASP factors (senomorphics) may provide a complimentary approach to senescence therapeutics such as senolytics and lifestyle adaptations.

Exposure to chemotherapeutic drugs such as Actinomycin D (ActD) can induce senescence in human mesenchymal stem cells (hMSCs) (153). Whilst the resident cells may be prevented from becoming cancerous, the effect of the SASP-generating senescent cells on the hMSC niche may be detrimental over time. Therefore, it is important that be able to target the induced senescent cell populations for removal, to give more tailored treatments.

Studies in which senescent cells were transplanted into healthy young mice, showed an increase in mortality as a result of senescent cell burden, which was subsequently reversed by intermittent administration of dasatinib and quercetin (154). This reduction of senescent cell burden has also been replicated in humans with diabetic kidney disease, however effects on mortality remain to be assessed (150). Elimination of senescent cells relieved persistent physiological dysfunctions including the secretion of frailty-related pro-inflammatory cytokines, as demonstrated by human adipose tissue explants (154). This and other methods are outlined in **Table 1**, adapted with permission (160).

**Table 1:** Experimental models of senescent cell deficiency/induction/depletion in the kidney and their effects on renal outcomes.

Reference	Model	Modulation of Senescence	Outcome	Effect of Any Intervention
(43)	Natural aging In INK-ATTAC mice	INK-ATTAC +AP20187 or vehicle administration to deplete p16^ink4a^+ cells	↑Glomerulosclerosis ↑ ß-gal positivity	↓Glomerulosclerosis ↓ß-gal positivity
(155)	Natural aging p16-3MR mice and fast aging Xpd TTD/TTD mice	FOXO4-DRI agent causes p53 nuclear exclusion. Ganciclovir Rx to p16-3MR mice causes p16^ink4a^+ restricted cell death	↑Serum Urea ↓Lmnb1 expression ↑SASP expression (both Xpd^TTD/TTD^ and aged p16-3MR)	FOXO4-DRI or GCV to p16-3MR admin: ↓Serum Urea ↑Lmnb1 expression ↓SASP expression (both Xpd^TTD/TTD^ and aged p16-3MR)
(34)	Nephrogenesis	WT *vs* P21^cip1^ KO mice with deficient growth arrest in nephrogenesis	↓ ß-gal positivity in P21^cip1^ KO mice utero. ↑Ki67 expression but ↑Apoptosis maintains development	Use of PI3K inhibitor augments developmental senescence in WT mice
(156)	UUO	WT *vs* p16^ink4a^ KO mice with impaired cell cycle arrest	UUO induces ß-gal positivity, apoptosis, and collagen deposition in WT mice	↓ ß-gal positivity ↓Apoptosis ↑Collagen, ↑proliferation after UUO in p16^ink4a^ KO
(157)	Renal IRI	WT *vs* P21^cip1^ KO mice with impaired cell cycle arrest	WT mice show tubular injury and raised blood urea levels after IRI	↑proliferation ↓Renal function ↑Mortality in P21^cip1^ KO
(156)	Renal IRI	WT *vs* p16^ink4a^/p19^ARF^ Double KO mice with impaired cell cycle arrest	WT mice show marked p16^ink4a^ and p19^ARF^ induction 28d after IRI, with apoptosis and reduced tubular density	p16^ink4a^ and p19^ARF^ deficient mice show improved epithelial and microvascular repair, with increased myeloid cell recruitment
(158) (159)	Diabetic Nephropathy	WT *vs* p21^cip1^ KO WT *vs* p27^kip1^ KO	WT mice develop albuminuria and glomerular hypertrophy	Both p27^kip1^ KO and p21^Cip1^ KO mice were protected from proteinuria and glomerular expansion
(52)	Renal Transplant	p16^ink4a^ KO mice with impaired cell cycle arrest	WT mice develop interstitial fibrosis and tubular atrophy	p16^ink4a^ KO mice develop less atrophy and fibrosis after Tx
(154)	Senescent cell transplant (young and older mice)	Dasatinib and Quercetin (D+Q) - senolytic administration	↑ Frailty ↑ Mortality ↑ Senescent burden	↓In frailty-related + pro-inflammatory cytokines (IL-6, IL-8, CCL2, PAI-1, GM-CSF) ↑ Survival
(150)	Diabetic Kidney Disease (Human)	Dasatinib and Quercetin (D+Q) - senolytic administration	↑ ↑ Probability of end-stage kidney failure ↑ Senescent burden	↓ p16^INK4A^ and p21^CIP1^ positive cells ↓ SASP (IL-1α, IL-2, IL-6, IL-9, MMP-2, MMP-9, MMP-12) ↓ CD68^+^ macrophages

Transgenic and genetic knockout mice have been used to study the impact of 1) deficiencies in the induction of senescence or 2) depletion of established senescent cells. Several of these models are summarized in this table, with description of the experimental model of renal disease used, the alteration in senescence induction employed and any alterations in renal disease outcomes. TTD/TTD, trichothiodystrophy/trichothiodystrophy; GCV, ganciclovir; WT, wild-type; KO, knock-out; UUO, unilateral ureteric obstruction; IRI, ischemia-reperfusion injury; Tx, Transplant; PAI-1, plasminogen activator inhibitor-1; GM-CSF, granulocyte macrophage colony-stimulating factor; IL-, Interleukin-; MMP-, Matrix Metalloprotease. Adapted with permission (160).

#### Lifestyle

Hall and colleagues showed that many p16^INK4a^ and SA-β-gal positive cells in the adipose tissue of mice may have been SAMs (senescent associated macrophages), attracted by senescent cells, and displaying pro-inflammatory (“M1”) phenotype (126) which has been shown to create a sterile inflammatory environment associated with obesity, as reviewed comprehensively (161). If adiposity affects senescent cell numbers, this could indicate that life-style choices (i.e. weight loss by calorie restriction and exercise) may successfully reduce numbers of macrophages secreting pro-inflammatory cytokines, by minimizing excess adipose tissue in response to exercise, showing positive feedback by reducing leptin (satiety hormone) (162) and minimizing adipose tissue – which is reported to contain large numbers of senescent cells (3) – and cause inflammation (126, 154). As briefly reviewed, by Xu and colleagues (163) pro-inflammatory mediators can also be reduced by CR. This may be a relevant therapeutic intervention due to the oxidative damage in response to transplantation procedures, caused by ischemia-reperfusion injury, which may lead to an increase in senescent burden and therefore premature ageing of the transplant and surrounding tissues (164). However, as previously mentioned this could lead to accelerated grey matter atrophy (18), meaning cost-benefit analysis needs to be considered for administration to patients.

## Therapeutics – Conclusions

The presence of senescent cells may be the most relevant case of the antagonistic pleiotropy theory of ageing (139), in that the key genes involved in senescence are beneficial in early life, promoting healthy embryogenesis (33) and rapid wound repair whilst protecting from cancer formation/progression (35). However, as senescent burden increases with age, SASP generating cells comprise a larger proportion of non-regenerating cell populations in tissues, negatively affecting homeostasis (42, 126, 165). This would suggest that certain therapeutic interventions, such as pharmaceutical treatment (senolytics/senomorphics) or lifestyle changes [regular aerobic exercise (3, 162)] may only show a significant impact when administered to patients of a certain age, who have accumulated enough senescent cell burden, and pharmaceutical interventions may be preventative. Senescence has wide-reaching effects and the elective administration of appropriate senolytics has the potential to improve the quality of life for patients with chronic conditions and age-associated morbidity, and further clinical trials are warranted.

## Challenges for Future Research Into Ageing-Associated Fibrosis

Ageing is difficult to model in a translationally significant manner using genetically close models, due to the marked expense and timescale limitations of non-human primate (NHP) models such as rhesus monkeys that live three to four decades. Although uncommon, these studies have been performed, pooling non-human primates (gorilla, rhesus monkey, baboon and others) to identify conservation of certain ageing phenotypes across species, such as arterial thickening correlated to increased age (166). The trans-NIH GSIG summit identified the induction of pathologies in young mice precludes analysis of interactions with other aspects of ageing seen in chronic diseases, requiring longer term studies to recapitulate the ageing phenotype before pathology induction (4). To overcome the limitations of relatively long-lived animals used for ageing, other vertebrate models have been assessed for use, for example the African turquoise killifish (*Nothobranchius furzeri*) has been assessed as a model for aging against the “Hallmarks of Ageing” (1) and has begun to be genetically altered and separate lines bred to provide a genetic toolkit to investigate ageing (167, 168).

## Final Conclusions

Senescent cells are attractive candidates as drivers of age-related organ dysfunction. They are consistently seen in diseased and older tissues when compared with healthy age-matched controls, actively secreting pro-inflammatory and pro-fibrotic molecules (90–92) capable of driving further (paracrine) senescence and propagating on-going tissue damage (78, 125). This is potentially because they secrete pro-inflammatory cytokines in the SASP which modify the surrounding environment (169).

Macrophages contribute to clearance of senescent cells by phagocytosis (73). This activity declines with age in multiple organ systems (115), including the kidney, as macrophages polarize from M1 to M2 in response to exogenous growth factors (170), and can potentially become ‘senescent-associated’ (78, 125, 133) and possibly senescent themselves (129). This is followed by a concurrent increase in fibrosis with age, which negatively affects organ function.

New therapy strategies have been developed, both pharmaceutical (39, 150) and lifestyle changes (126, 162) that aim at reducing the burden of senescent cells and the SASP they generate, and reducing inflammation, aimed at removing blockades for macrophage polarity transitions essential for response to injuries. This research is slowed by the feasibility of ageing models currently utilized, however efforts by researchers have brought new animals more relevant to ageing research into mainstream use, as is the case with the African Killifish (167, 168).

Ageing is a complex interaction of different physiological responses that can be influenced by multiple factors from genetics to the environment, but current research has and is investigating these interactions and different factors for therapeutic benefit in humans.

## Author Contributions

RC: First author and primary writer. M-HD: Content contributor. DF: Content contributor and editor. KJM: Content contributor, editor, and last author. All authors contributed to the article and approved the submitted version.

## Funding

RC is supported by the Wellcome Trust [108906/Z/15/Z]. M-HD is supported by a MRC Clinical Training Research Fellowship (CRTF) [MR/T008253/1]. DF is supported by Intermediate Clinical Fellowship 1243 [WT100171MA] from the Wellcome Trust. KJM is supported by a Chief Scientist Office/Kidney Research UK Fellowship [CSO_PDF/2018/1].

## Conflict of Interest

The authors declare that the research was conducted in the absence of any commercial or financial relationships that could be construed as a potential conflict of interest.
